# Invariant correlation: a general and straightforward method to quantify invariance in time-varying neural signals

**DOI:** 10.32470/srbsjd0

**Published:** 2026

**Authors:** Jarrod M. Hicks, Dana Boebinger, Kirill V. Nourski, Matthew A. Howard, Christopher M. Garcia, Thomas Wychowski, Webster Pilcher, Sam V. Norman-Haignere

**Affiliations:** 1 Department of Neuroscience, University of Rochester Medical Center; 2 Department of Biostatistics & Computational Biology, University of Rochester Medical Center; 3 Department of Neurosurgery, University of Iowa; 4 Iowa Neuroscience Institute, University of Iowa; 5 Department of Neurology, University of Rochester Medical Center; 6 Department of Neurosurgery, University of Rochester Medical Center; 7 Department of Brain & Cognitive Sciences, University of Rochester; 8 Department of Biomedical Engineering, University of Rochester

## Abstract

Recognizing information in natural stimuli is challenging because sensory inputs for different instances of the same feature often vary substantially. Successful recognition thus requires neural systems to generate representations that are invariant to such variation, posing a fundamental computational challenge for sensory coding. Much remains unknown about how sensory systems code invariant information in complex, temporally dynamic stimuli such as speech, in part due to methodological challenges in measuring and modeling invariance from noisy, time-varying neural responses. Here, we introduce the “invariant correlation”, a straightforward and general method for directly quantifying the strength and temporal dynamics of invariance from time-varying neural signals and computational models. We demonstrate its utility by quantifying invariance to acoustic variation in neural representations of phonemes using spatiotemporally precise intracranial recordings from human auditory cortex. Our method successfully revealed substantial diversity in the strength and dynamics of invariance across the human auditory cortex and across different phonetic features within individual electrodes. We illustrate how many of these empirically measured patterns can be explained by applying the same analysis to the predictions from a simple computational model based on spectrotemporal tuning in a cochleagram representation of sound. Although we focus here on speech, the invariant correlation is a broadly applicable tool for characterizing the organization and computational mechanisms underlying invariant representations of dynamic stimuli across sensory modalities.

## Introduction

Recognizing information in natural stimuli is challenging because different instances of the same feature differ substantially in their sensory characteristics. For example, two instances of the same phoneme can have vastly different acoustic properties depending on the speaker and context in which the phoneme is produced (Allen et al., 2003; Blumstein and Stevens, 1979; Hillenbrand et al., 1995; Ladefoged and Broadbent, 1957; Peterson and Barney, 1952; Stevens and Weismer, 2001). Recognition thus requires neural systems to code information in a way that is invariant to or tolerant of identity-preserving changes in the sensory signal (DiCarlo and Cox, 2007; DiCarlo et al., 2012).

Many important questions remain unanswered about the neural computations that support tolerance to sensory variation, particularly for dynamic stimuli such as speech (Bizley and Cohen, 2013). Progress has been limited in part by methodological challenges associated with measuring and modeling invariance from noisy, time-varying neural data. To address these challenges, we introduce the invariant correlation, a straightforward analysis method to directly quantify invariance in time-varying neural signals from any sensory domain (e.g., audition, vision) or time-resolved neural recording (e.g., single/multiunits, EEG, MEG).

Our method provides a continuous, time-varying measure that reveals both the strength and temporal dynamics of invariance for any sensory feature (e.g., phonemes, words, or pitch) present in natural stimuli. It incorporates an explicit noise ceiling to account for neural recording noise, enabling comparisons across regions and participants that differ in reliability, and can be directly applied to predictions from computational models, enabling close brain-model comparisons.

We demonstrate this method by characterizing invariant representations of phonemes in speech, using spatiotemporally precise intracranial recordings from neurosurgical patients. Phonemes unfold on rapid timescales, and different utterances of the same phoneme can differ substantially in their acoustic realization (Hillenbrand et al., 1995; Liberman et al., 1967), making them an ideal test bed for probing invariant neural representations. Yet despite extensive research studying phoneme encoding in the human auditory cortex (Chang et al., 2010; Mai et al., 2024; Mesgarani et al., 2014), no studies have directly quantified tolerance to acoustic variation in cortical representations of phonemes while accounting for neural noise or directly tested what computational transformations underlie such tolerance. Using our method, we reveal diversity in the timing, strength, and pattern of invariance both across electrodes and across phonetic features (e.g., plosives, fricatives, etc.), and then show how applying the same analysis to the predictions of a simple acoustic model helps to explain this diversity.

## The invariant correlation

The core logic of our method is simple: if a neural representation of a stimulus feature is invariant, then it will produce the same response whenever that feature is present across all other stimulus variation. Thus, we can measure invariance by quantifying the similarity of neural responses across different instances of stimuli that share the same feature. Importantly, this requires specifying both the feature of interest (i.e., what is being represented) and the set of stimulus instances across which that feature is compared. We measure invariance using the invariant correlation, which makes explicit the comparison set across which invariance is evaluated. We illustrate our approach using intracranial recordings from human auditory cortex to measure invariance to acoustic variation in neural representations of phonemes (Fig. 1). However, we emphasize that our method can be used to characterize invariance for any stimulus feature (e.g., phonemes, words, pitch) using neural or model responses to any natural, time-varying stimulus (e.g., speech, video).

Computing the invariant correlation involves five steps. First, neural activity is measured in response to stimuli containing variation. In our case, participants listened to a diverse set of sentences spoken by different talkers while neural responses were measured using intracranial EEG (see Appendix). Second, the neural response time course is segmented into response windows surrounding individual instances of the feature of interest. While our analysis can be applied to any stimulus feature or structure, here we focus on phonemes, because they are a canonical speech unit with substantial acoustic variability (Hillenbrand et al., 1995; Stevens and Weismer, 2001). We chose to align segments by feature onset, though we repeated our analyses aligning to phoneme offset and found that the results are robust to alignment choice (Figs. A1–2). Third, these segments are assembled into a response matrix $\text{R}\in R^{N\times T}$ in which each row corresponds to one of N feature instances and each column corresponds to one of T time points relative to feature onset. Fourth, a second response matrix ${\text{R}}_{\pi }$ is created by permuting the rows of the original matrix to preserve feature values while varying other stimulus dimensions. This permutation defines the comparison set and thus specifies the variation across which invariance is evaluated. In our case, each phoneme instance is paired with a different instance of the same phoneme identity that differs in acoustics (i.e., spoken by a different talker and occurring in a different word). Finally, we compute the invariant correlation $\text{IC}(t)=\text{corr}\left(R(:,t),R_{\pi }(:,t)\right)$ by correlating the two columns of the response matrices at time t.

Because the invariant correlation is computed separately at each time point relative to feature onset, this analysis produces a time course reflecting the extent of invariance at each moment in time. Prior to feature onset, the responses being correlated are driven primarily by stimulus segments for which the feature of interest is not explicitly matched across paired instances. Following feature onset, the neural response becomes increasingly influenced by matched feature, causing the correlation to rise if neural activity reflects an invariant response to the shared feature. As time progresses beyond the offset of the matched segments, the response again becomes dominated by unmatched feature values, causing the correlation to decrease. Finally, we note, because the invariant correlation is computed across feature instances, it is sensitive only to response patterns that vary systematically across instances and is insensitive to responses shared uniformly across all instances (e.g., a generic onset response).

Importantly, the magnitude of the invariant correlation is limited by noise in the neural recordings, such that even fully invariant responses will not yield a correlation of 1. To account for this, we also estimate a noise ceiling reflecting the maximum correlation achievable, given the measurement noise in the neural recordings. The noise ceiling is computed using the same response matrix and correlation procedure described above, but instead of pairing different feature instances, we pair responses across repeated presentations of identical feature instances (i.e., the same phoneme occurring in the same word spoken by the same talker). Formally, at each time point $t,\text{NC}(t)=\text{corr}\left(R(:,t),R_{\text{rep}}(:,t)\right)$, where $R_{\text{rep}}$ pairs repeated presentations of identical feature instances. In the absence of noise, this correlation is expected to be 1. Deviations from this value, therefore, reflect attenuation due to noise and thus provide an upper bound on the maximum achievable correlation. Dividing the invariant correlation by the noise ceiling therefore implements the classical correction for attenuation due to measurement noise (Spearman, 1904). To validate this normalization, we ran simulations using synthetic data with known levels of invariance and confirmed that our procedure accurately recovered the ground-truth invariance across noise levels (Fig. A3). As with the invariant correlation, this computation is performed separately at each time point relative to feature onset, yielding a noise ceiling time course. However, because both feature identity and surrounding context are matched across repetitions, the noise ceiling is expected to be relatively stable over time, rather than exhibiting the dynamics observed for the invariant correlation. A more formal treatment of the invariant correlation and noise correction can be found in the Appendix.

## Application of the invariant correlation

### Measuring the strength and timing of invariance across human auditory cortex

To illustrate our method, we applied it to characterize invariant representations of phonemes across 183 sound-responsive electrodes from 25 patients with extensive coverage of both primary and non-primary auditory cortex. Figure 2A shows the invariant correlation and noise ceiling for several example electrodes, illustrating the diversity of patterns revealed by our method. Many electrodes showed evidence for partial invariance to acoustic variation, with some exhibiting rapid, transient peaks in the invariant correlation following phoneme onset (e.g., E1), and others showing more delayed and sustained invariance (e.g., E2). We also observed electrodes with near-zero invariance, despite a non-zero noise ceiling (e.g., E3). Importantly, a non-zero noise ceiling reflects reliable, differential responses across phonemes, while a near-zero invariant correlation indicates that those responses are inconsistent across different instances of the same phoneme. Thus, our method provides a continuous measure of invariance strength and timing that makes it possible to make affirmative statements about the absence of invariance, as well as the (graded) presence of invariance.

We can obtain a concise and interpretable summary of invariance using the peak of the invariant correlation time course. The peak value divided by the noise ceiling at the corresponding time point provides a measure of the invariance strength. The timing of the peak provides a measure of latency. Because the latency is only meaningful if there is a non-zero peak, we discarded electrodes with a non-significant peak value (see Appendix). Both measures were highly reliable across data splits (r=0.92 for both). These scalar metrics enable investigating the anatomical distribution of invariance strength and latency (Fig. 2B–C) and quantifying anatomical organization using standard regions of interest (ROIs) (Fig. 2D–F). Invariance strength showed substantial variability across electrodes (median=0.13, IQR=0.08-0.18). However, values were nearly exclusively below 0.4, indicating that most of the reliable variance to phonemes is not consistent across acoustic variation. We also did not observe any clear spatial clustering of invariance, with all anatomical regions showing a similar diverse range of invariance strengths. Invariance latency also showed substantial heterogeneity (median=70ms, IQR=60-110ms), but exhibited clearer anatomical organization with longer latencies in more non-primary regions, particularly in the superior temporal gyrus (STG) (Fig. 2F). Collectively, these analyses illustrate how our method can be used to characterize invariance across human auditory cortex. The mechanisms underlying these empirical patterns can then be investigated by comparing the measured results with predictions from computational models, as we later demonstrate.

### Identifying feature-specific patterns of invariance within individual electrodes

An important feature of our method is that it can be flexibly applied to different stimulus features, enabling more granular analyses of invariance. To demonstrate this, we repeated our analysis for subsets of phonemes that share a phonetic feature (e.g., plosives). Specifically, for each electrode, we measured the invariance strength for phonemes sharing one of 19 phonetic features (Hamilton et al., 2021; Keshishian et al., 2023; Mesgarani et al., 2014), yielding a phonetic-feature-specific pattern of invariance. Because these analyses are based on subsets of the full stimulus set, we first assessed the reliability of these patterns at the level of individual electrodes. Figure 3A shows the split-half reliability across electrodes (median=0.54, IQR=0.39–0.70), indicating that phonetic-feature-specific patterns of invariance were generally stable. Figure 3B shows the correspondence between split-half estimates of the invariance pattern and illustrates the variation in invariance strength across phonetic features within a single, highly reliable electrode.

Inspection of individual electrodes revealed that invariance differed substantially across phonetic features (Fig. 3C–E). To illustrate this, we focus on phonetic dimensions such as manner of articulation, place of articulation, and vowel backness because they provide well-established partitions of phonemes that have been widely used to probe structure in auditory cortical representations of speech (Hamilton et al., 2021; Keshishian et al., 2023; Mesgarani et al., 2014; Yi et al., 2019). For example, one electrode shown in Figure 3C (E1) showed partially invariant responses for plosives but not fricatives, while another (E2) exhibited the opposite pattern. Similar contrasts were observed for vowel backness (Fig. 3D), with one electrode (E3) showing greater invariance for front vowels compared to back vowels, and another (E4) exhibiting the reverse pattern. For place of articulation (Fig. 3E), different electrodes preferentially exhibited invariance for coronal (E5), dorsal (E6), or labial (E7) consonants. Together, these examples illustrate how applying our method to different features can successfully reveal structured patterns of invariance within individual electrodes.

### Evaluating invariance in computational models

The results described above reveal substantial variation in the patterns of invariance across human auditory cortex, raising the question of whether these patterns reflect differences in underlying neural computations. Because the invariance analysis can be flexibly applied to any stimulus-response time course while accounting for noise, it can be applied to model-predicted responses, enabling direct, quantitative comparisons between neural invariance and that of computational models.

To illustrate this approach, we applied our invariance analysis to a standard spectrotemporal receptive field (STRF) model in which neural responses are characterized by linear tuning for patterns of spectrotemporal modulation in a time-frequency representation of sound (Hullett et al., 2016; Meyer et al., 2017; Norman-Haignere et al., 2022; Sadagopan et al., 2023; Thorson et al., 2015; Woolley et al., 2005). While we note that this method could be applied to any computational model, such as deep neural network models, we focus on a STRF model here to test whether a simple, commonly-used acoustic model could explain our empirical results. Specifically, we first fit STRF encoding models to each electrode (Fig. 4A) using standard methods (ridge regression with nested cross-validation; see Appendix). After fitting each model on training data, we then measured invariance in the model-predicted responses to held-out speech stimuli using the same procedure we applied to the neural recordings. As a baseline, we also repeated this analysis using a simpler waveform envelope temporal receptive field (TRF) model that predicts neural responses from sound energy alone (see Appendix; Fig. A4).

We found that even a simple STRF replicated many empirical patterns observed in the data. Across electrodes, the invariance computed from STRF model predictions tracked that observed in the neural data (Fig. 4B). Both invariance strength and latency were strongly correlated between the data and model across electrodes (Fig. 4C; strength: r=0.83, p<0.001 hierarchically bootstrapped 95% CI: [0.53, 0.83]; latency: r=0.78, p<0.001, hierarchically bootstrapped 95% CI: [0.43, 0.82]). Within individual electrodes, the model also reproduced relative differences in invariance strength across phonetic features for many electrodes (Fig. 4D–F). Interestingly, we found that the overall invariance strength was slightly higher for the model than for the neural data. Since our measures are noise-corrected, this reflects a genuine mismatch between the neural data and the model predictions, rather than differences in reliability. A simpler waveform-envelope model performed significantly worse than the STRF model (hierarchically bootstrapped 95% CI for difference in brain–model correlations between models: [0.11, 0.57]; Fig. A4), indicating that the observed invariance cannot be explained by generic responses to sound energy alone, but instead reflects structured variation in neural responses across phoneme categories. Together, these analyses illustrate how our method can be used alongside modeling to evaluate hypotheses about which computational mechanisms can account for observed patterns of invariance in neural data.

## Discussion

### Summary of contributions

We introduce a general method for quantifying the strength and temporal dynamics of invariance in neural responses and computational models. Our method provides a continuous and interpretable measure of invariance, along with a noise ceiling, allowing values to be interpreted on a common scale and compared across recording sites. The method is model-free, not relying on assumptions about the stimulus-response mapping (e.g., encoding models) or readout scheme (e.g., decoding models). Because our method operates directly on stimulus-response time courses, it can be applied across recording modalities and stimulus domains. The same analysis can also be applied to model-derived responses, enabling direct model–brain comparisons to assess whether specific computational models reproduce the patterns of invariance observed in neural data.

We illustrated this method using spatiotemporally precise intracranial recordings from human auditory cortex, examining invariance to acoustic variation in neural representations of phonemes. Our method revealed heterogeneity in invariance across auditory cortex, with individual electrodes showing distinct patterns of invariance across phonetic features. We then applied our analysis to a STRF encoding model and found that it reproduced many empirical patterns, suggesting that much of the observed structure may reflect differences in underlying spectrotemporal tuning. Together, these results demonstrate how our method can characterize representational invariance and evaluate hypotheses about the computations that give rise to it. Although we focus on speech and phonetic structure, our method is general and provides a flexible tool for probing invariance across sensory modalities, stimulus dimensions, and computational models.

### Relation to prior methods

Decoding has been frequently used to study neural invariance by learning an explicit mapping that can reliably identify and separate features of interest from population responses (Bonte et al., 2014; Feng et al., 2018; Formisano et al., 2008; Gennari et al., 2021; Gwilliams et al., 2022; Hung et al., 2005; Isik et al., 2014; Okada et al., 2010; Rust and DiCarlo, 2010). Invariance is then typically inferred from classification accuracy in left-out test data. However, classification accuracy does not explicitly quantify the extent of variability in the neural responses and is highly sensitive to noise in the neural measurements (even with cross-validation). Cross-decoding partially addresses noise-related confounds by training a decoder on one stimulus condition and testing it on another, with successful generalization interpreted as evidence for invariance. Nonetheless, this approach infers invariance implicitly and its application is mostly limited to population-level analyses. Moreover, decoding-based methods require both choosing the form of the decoding model and obtaining enough training data to train the model, which typically requires impractically large numbers of trials for decoding continuous speech. In contrast, our method is model-free, requires no training data, and can be applied to neural recordings from individual sites to directly quantify invariance while correcting for measurement noise. Consequently, our approach can be used to compare invariance across different brain regions with different levels of noise, and to directly compare neural recordings with predictions from computational models.

A strength, and potential limitation, of our method is that it focuses on characterizing invariance at the level of individual recording sites rather than at the population level. Nonetheless, modest invariance at single electrodes could combine into stronger invariance in population-level representations, and our approach can be easily extended and combined with existing multivariate methods to study invariance in neural populations. For example, a decoder could be used to learn explicit projections of population activity that optimally separate phoneme categories. These projections are themselves time-varying signals, and thus the invariant correlation could be applied to the projections in the same way one would measure invariance from individual electrodes (see Appendix). The invariant correlation could also be naturally extended to explicitly capture invariance at the population level by substituting the Pearson correlation for a multivariate similarity measure (Robert and Escoufier, 1976).

### Relation to prior research on phoneme encoding

Neural representations of phonemes have been extensively studied using encoding models that predict neural responses with categorical regressors based on phoneme identity or phonetic features (de Heer et al., 2017; Di Liberto et al., 2015; Gong et al., 2023; Hamilton et al., 2021; Keshishian et al., 2023; Mesgarani et al., 2014). Because these models often explain additional variance relative to acoustic baseline models, prior studies have suggested categorical phoneme representations in some auditory cortical regions, such as the STG (Chang et al., 2010; Hamilton et al., 2021). Given that categorical representations are by definition invariant to acoustic detail, this interpretation implicitly suggests invariance in neural responses, seemingly contrasting with our findings of partial invariance. However, these studies have not explicitly measured tolerance to acoustic variation, and the variance explained by categorical regressors is often modest, consistent with our results. In addition, the apparent contribution of categorical regressors depends on the acoustic baseline used, which varies across studies and is sometimes relatively simple (e.g., the waveform envelope) (Leonard et al., 2024). When richer spectrotemporal tuning is accounted for, the unique variance explained by categorical regressors is often small (Daube et al., 2019), suggesting a more limited contribution of categorical representations than comparisons to simpler baselines imply.

The method we describe addresses these issues by explicitly quantifying invariance and applying the same analysis to predictions from computational models. This approach separates the experimental quantity of interest (invariance) from the choice of model (e.g., a STRF), allowing direct assessment of how well different models account for the data. Using this approach, we find that invariance in phoneme representations can, to a non-trivial extent, be explained by relatively simple STRF models. However, the STRF models do not fully account for all observed patterns of invariance. We emphasize that our goal is not to make definitive claims about the mechanisms underlying invariant representations of phonemes, but to introduce a general method for directly quantifying invariance, which we hope will be useful for studying invariant representations of many different time-varying features, including phonemes, and understanding the computational mechanisms that support them.

The modest invariance strengths observed for phonemes might seem puzzling given that, perceptually, listeners can reliably recognize speech across utterances and speakers. Although an in-depth scientific investigation of the link between neural and perceptual invariance is beyond the scope of this paper, discussing some of the possible explanations for weak invariance is illustrative of how our method can be combined with other approaches. One possibility is that the representational units underlying speech recognition are not well aligned with phonemes. For example, speech recognition might rely on diphones (Gong et al., 2023) or words (Zhang et al., 2026), which could be tested by measuring invariance for these units using the same analysis. Another possibility is that invariance is achieved by population codes, which could be tested by applying the invariant correlation to decoder-derived dimensions, as described above. The patterns could then be compared against computational models to determine whether there are patterns that cannot be explained by spectrotemporal tuning, but which can be accounted for by models that are explicitly optimized for invariant recognition (e.g., deep neural networks) (Kell et al., 2018; Feather et al., 2023, Yamins and DiCarlo, 2016).

### Methodological considerations, limitations, and future directions

Because our method measures invariance from time-resolved responses, differences in the timing at which neural representations become invariant across different stimulus instances could reduce the measured invariance. However, we note that this is a challenge faced by any kind of analysis that relies on temporal alignment, including encoding and decoding models (e.g., temporal jitter would decrease decoding performance). A benefit of the invariant correlation is that alignment is made explicit, and it is therefore straightforward to test different alignment methods. As an example, we repeated our analyses aligning responses to phoneme offset rather than onset and found that the results are largely unchanged (see Figs. A1–2), demonstrating that the metric is robust to reasonable choices of alignment, at least when the segments correspond to phonemes. Importantly, our method provides a framework for testing hypotheses about the temporal reference points to which neural computations are aligned and about how invariant representations emerge over time. Exploring these dynamics, especially in stimuli with variable temporal structure like natural speech, will be an important direction for future research.

Here, we quantified response similarity using Pearson correlation, which provides a bounded and interpretable measure of response consistency. However, the general approach to measuring invariance is not tied to any specific similarity metric, and correlation could be replaced with alternative similarity measures depending on the domain and signal properties. Future work could systematically examine how different similarity measures influence estimates of invariance and whether they reveal additional structure beyond that captured by correlation.

Because the invariant correlation quantifies similarity between neural responses to paired instances of the same feature, it is necessarily conditioned on the relationship between those instances. In other words, the comparison set used to measure invariance determines the distribution of variation across which invariance is assessed, making the choice of comparison set an important methodological consideration. Thus, the modest invariance values observed for individual electrodes reflect the specific variation tested rather than a limitation of the method, and still provide meaningful insights. In the present study, phoneme instances were paired across different words spoken by different talkers. However, invariance estimates would differ under alternative comparison sets. For example, pairing phoneme instances produced by the same speaker would reduce acoustic variability and would likely increase invariance, whereas deliberately pairing instances to maximize acoustic dissimilarity (e.g., across speakers with markedly different vocal tract characteristics) would likely decrease it. This means that invariance should not be considered a fixed quantity but must be interpreted relative to the specific variation tested. Our method makes it straightforward to quantify invariance across different types of variation simply by changing which stimulus instances are paired during the calculation of the invariant correlation. This flexibility thus provides a means to test hypotheses about invariance across different types of variation and then assess whether computational models can explain the resulting patterns of invariance.

Another important feature of our method is that it is agnostic to the form of invariance in neural representations. The invariant correlation quantifies the similarity of responses across different instances of a feature without specifying the structure of the underlying representation. For example, apparent invariance for phonemes could arise from a categorical representation of a phonetic feature (e.g., fricatives) or from a representation that distinguishes individual phonemes. However, because our method can be flexibly applied across stimulus features, it supports iterative exploration of which features drive the observed invariance. This flexibility enables systematic discovery of the relevant features and granularity at which invariance is expressed in neural representations.

## Figures and Tables

**Figure 1:**
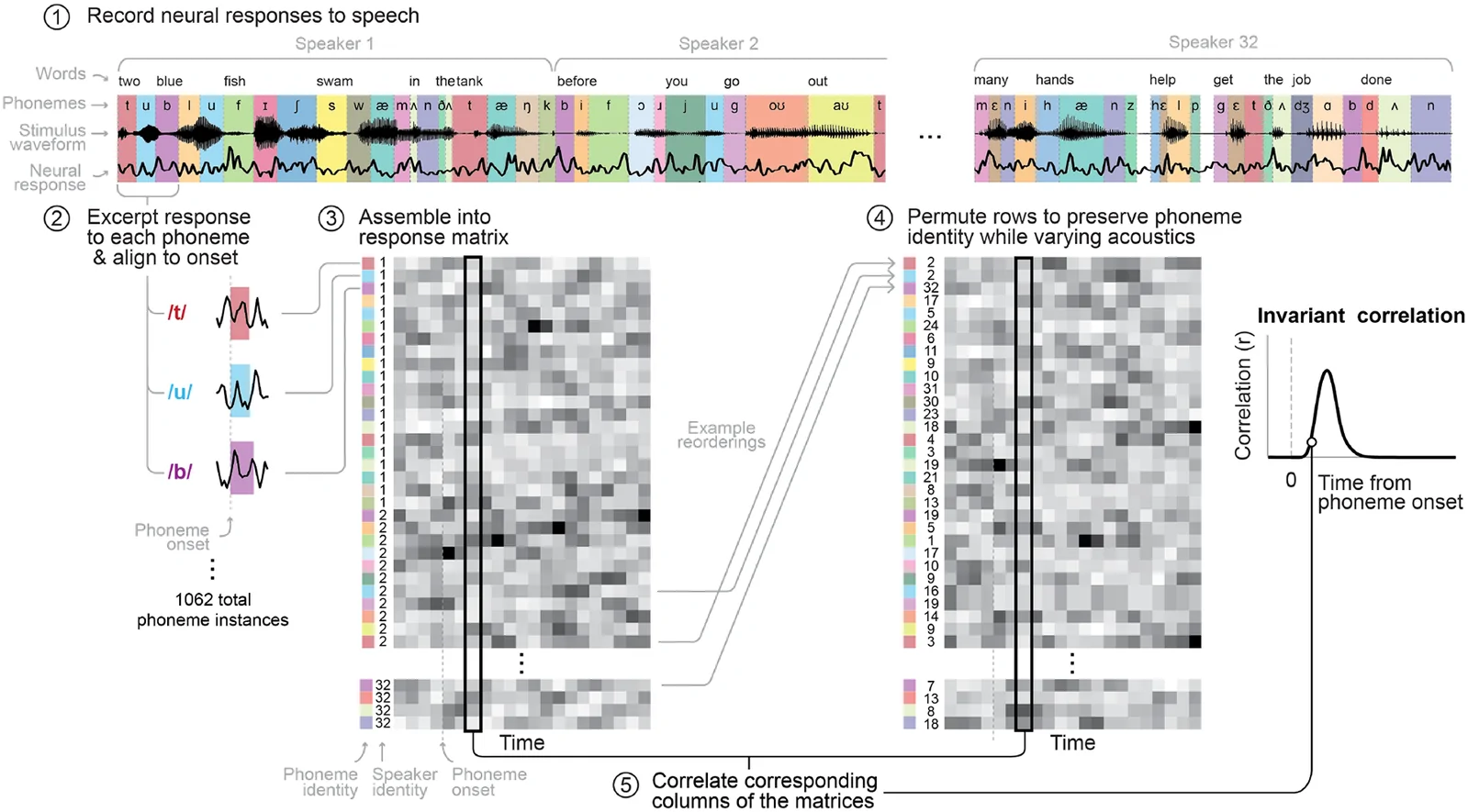
Schematic of the invariant correlation. **(1)** Record neural responses to speech with natural acoustic variation (sentences spoken by different talkers). **(2)** Segment neural response time course around individual phonemes and align to phoneme onset. **(3)** Assemble segments into a time-resolved response matrix with rows corresponding to individual phoneme instances and columns corresponding to time relative to onset. **(4)** Create a second response matrix by permuting the rows to pair each phoneme instance with a different instance of the same phoneme identity that differs in acoustics. **(5)** Compute the invariant correlation by correlating corresponding columns of the two matrices to measure similarity of responses to phonemes that share identity but vary acoustically.

**Figure 2:**
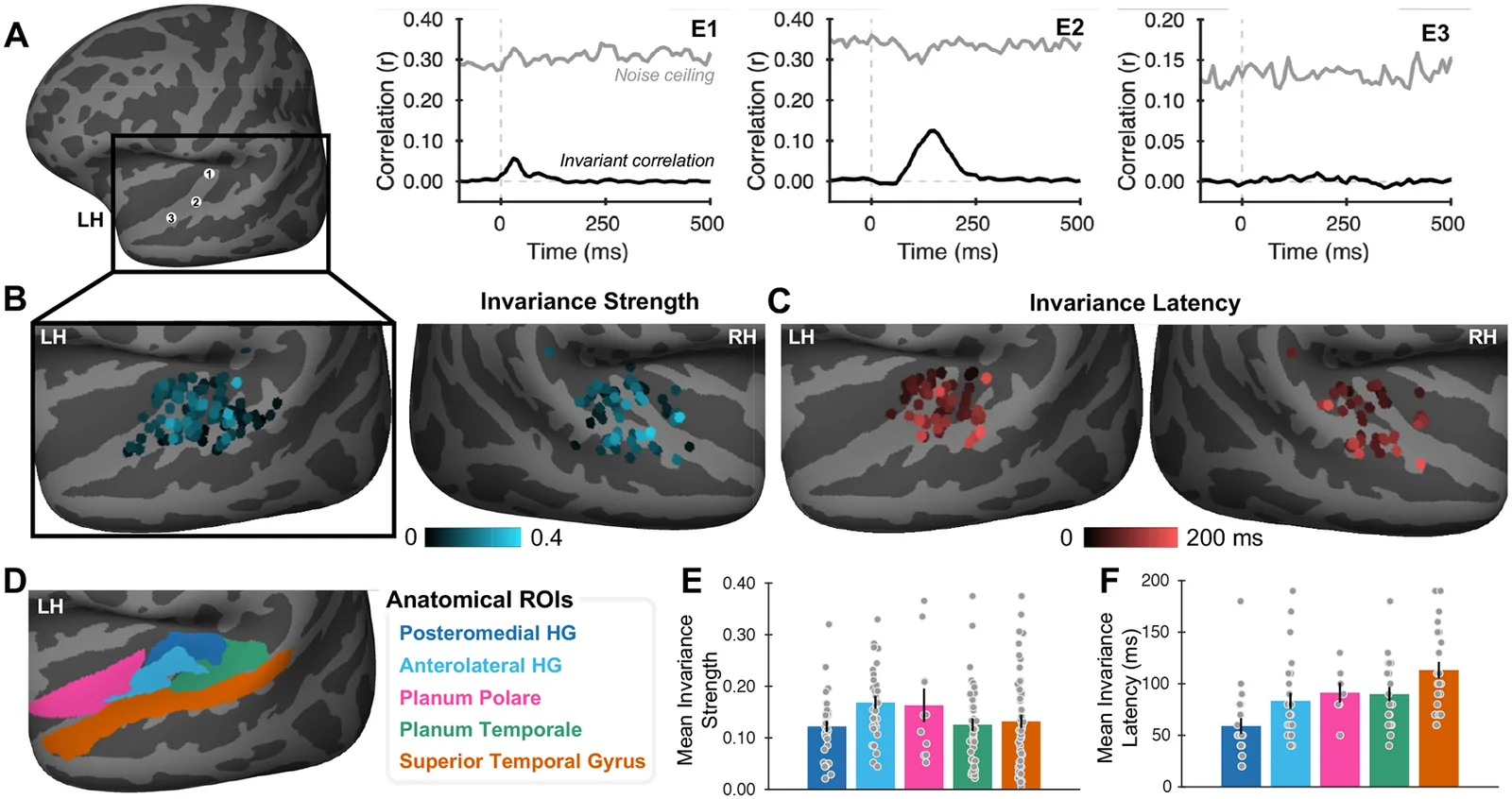
Strength and timing of invariance across human auditory cortex. **(A)** Invariant correlation (black) and noise ceiling (gray) for example electrodes. **(B)** Cortical map of invariance strength for each electrode, defined as the peak invariant correlation divided by the noise ceiling at that latency. **(C)** Cortical map of invariance latency, defined as the time of the peak invariant correlation. **(D)** Anatomical ROIs; HG = Heschl’s Gyrus. **(E)** Mean invariance strength across different ROIs. Dots = electrodes; bars = SEM. **(F)** Mean invariance latency across different ROIs. Same conventions as (E).

**Figure 3:**
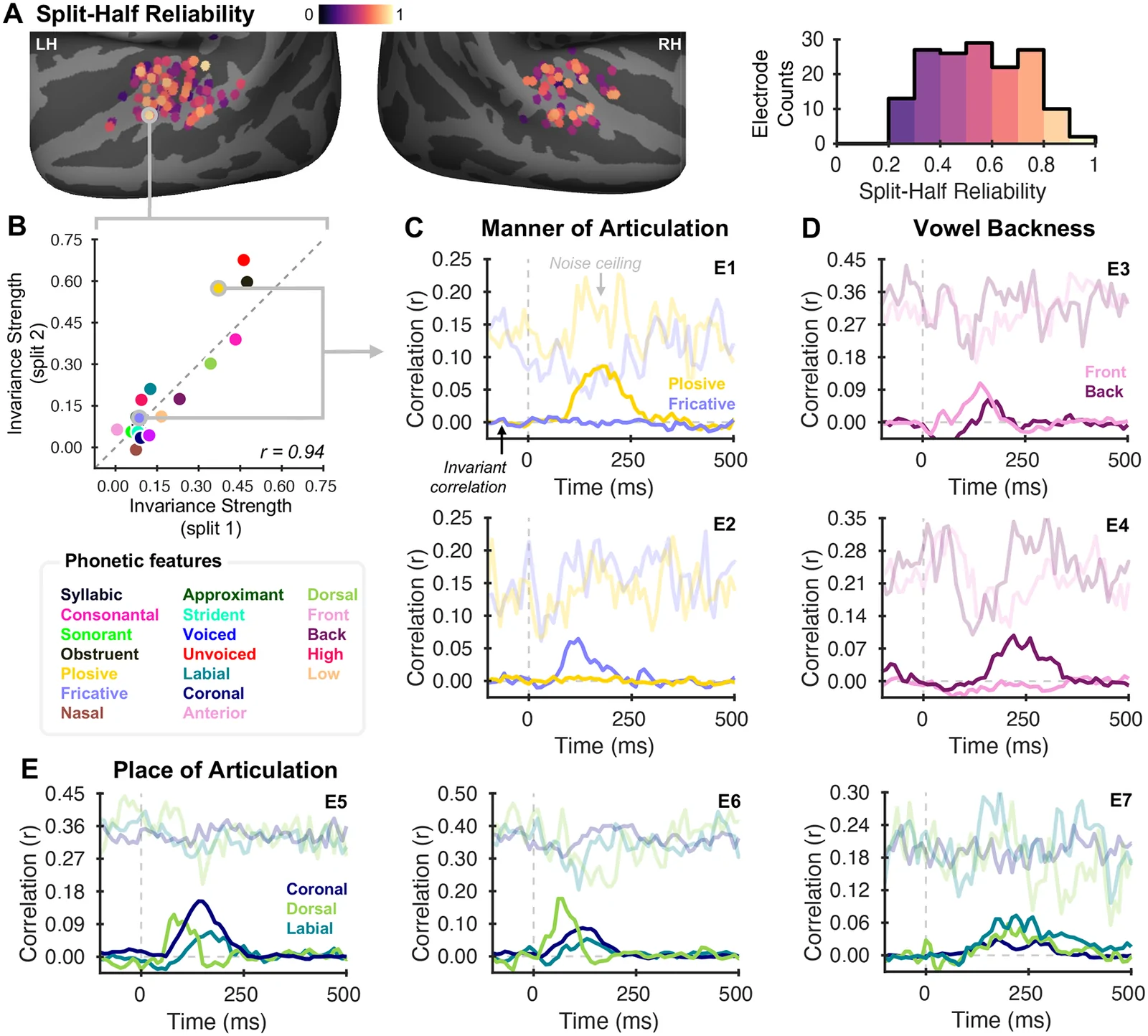
Phonetic-feature-specific invariance. **(A)** Cortical map of split-half reliability of invariance pattern across phonetic features for each electrode; right shows distribution across electrodes. **(B)** Correspondence between split-half estimates of the phonetic-feature-specific invariance pattern for an example electrode. Each point reflects invariance strength for one phonetic feature estimated from independent splits. **(C)** Invariant correlation (saturated) and noise ceiling (desaturated) time courses for example electrodes showing differential responses for phonetic features related to manner of articulation **(D)** Same as (C), for vowel backness. **(E)** Same as (C), for place of articulation.

**Figure 4:**
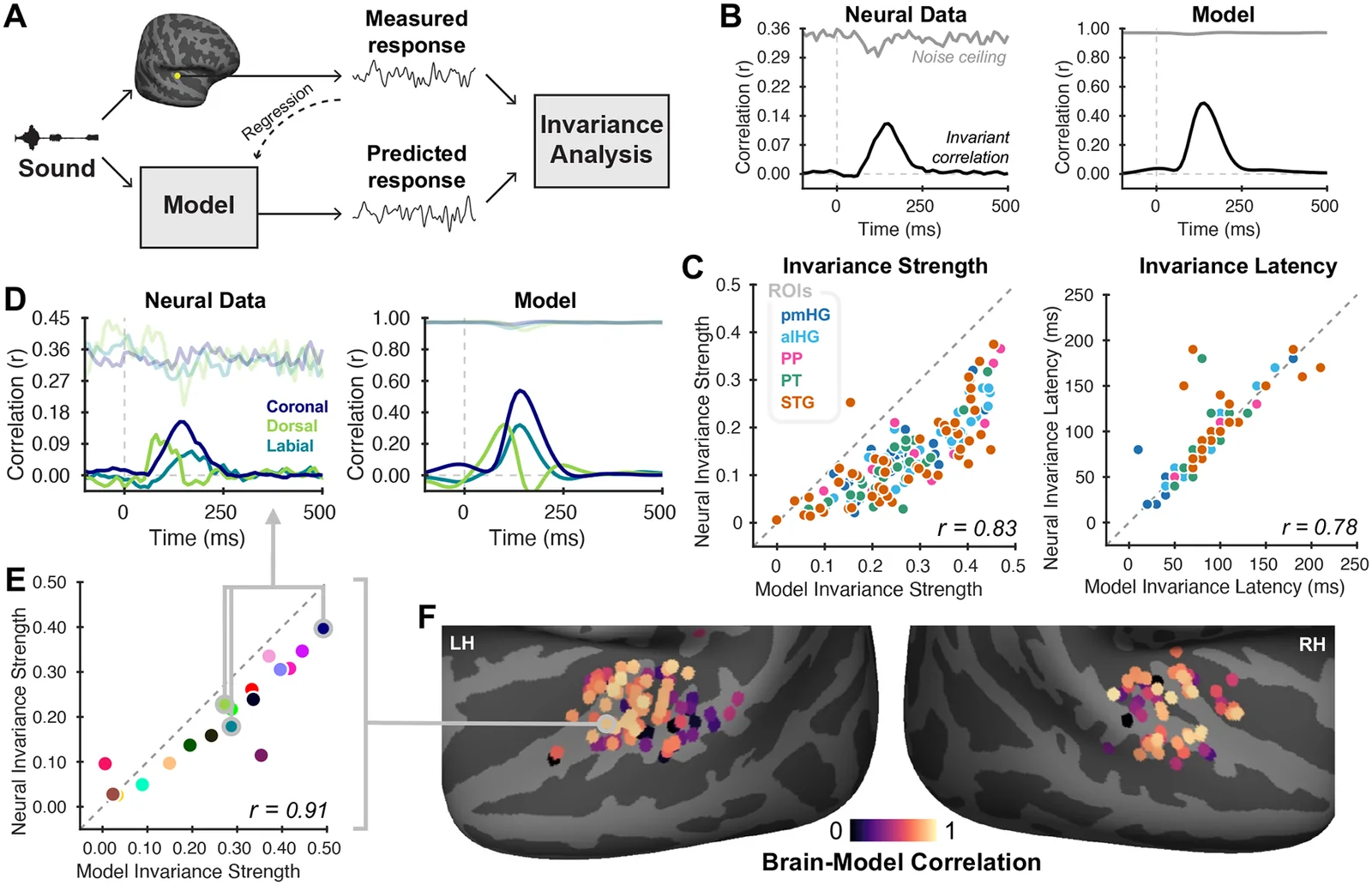
Comparing invariance in computational models and neural data. **(A)** Schematic illustrating application of the method to computational models: an encoding model is fit to neural data, and invariance is measured from model-predicted responses to held-out stimuli using the same procedure as for neural recordings. **(B)** Example invariant correlation (black) and noise ceiling (gray) for one electrode measured from neural data (left) and STRF model predictions (right). **(C)** Left, invariance strength measured from STRF model predictions vs. neural data across electrodes (colored by anatomical ROI). Right, same comparison for invariance latency. **(D)** Example invariant correlations (saturated) and noise ceilings (desaturated) for phonetic features related to place of articulation for one electrode, measured from neural data (left) and STRF model predictions (right). **(E)** Correspondence between phonetic-feature-specific invariance patterns measured from neural data and STRF model predictions for one electrode. **(F)** Cortical map showing brain-model correlation of phonetic-feature-specific invariance patterns for each electrode.

**Figure A1:**
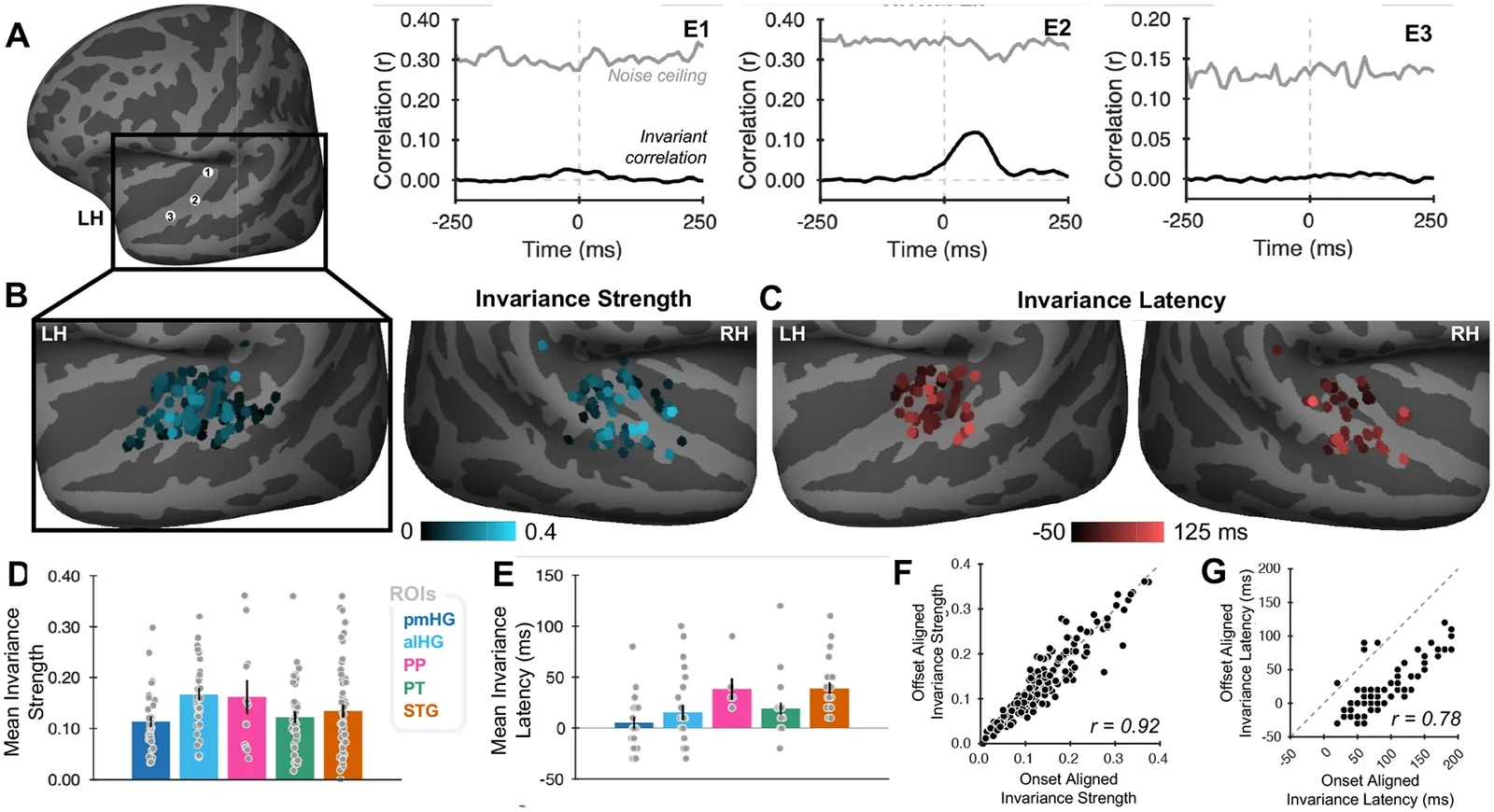
Strength and timing of invariance across human auditory cortex using responses aligned to phoneme offset. **(A)** Invariant correlation (black) and noise ceiling (gray) for example electrodes. Note timing is relative to phoneme offset. **(B)** Cortical map of invariance strength for each electrode, defined as the peak invariant correlation divided by the noise ceiling at that latency. **(C)** Cortical map of invariance latency, defined as the time of the peak invariant correlation. **(D)** Mean invariance strength by ROI. Dots = electrodes; bars = SEM. **(E)** Mean invariance latency by ROI. Same conventions as (D). **(F)** Comparison of invariance strength across alignment choices. Each dot is an electrode. Gray dashed line represents identity line. **(G)** Comparison of invariance latency across alignment choices. Same conventions as (F).

**Figure A2:**
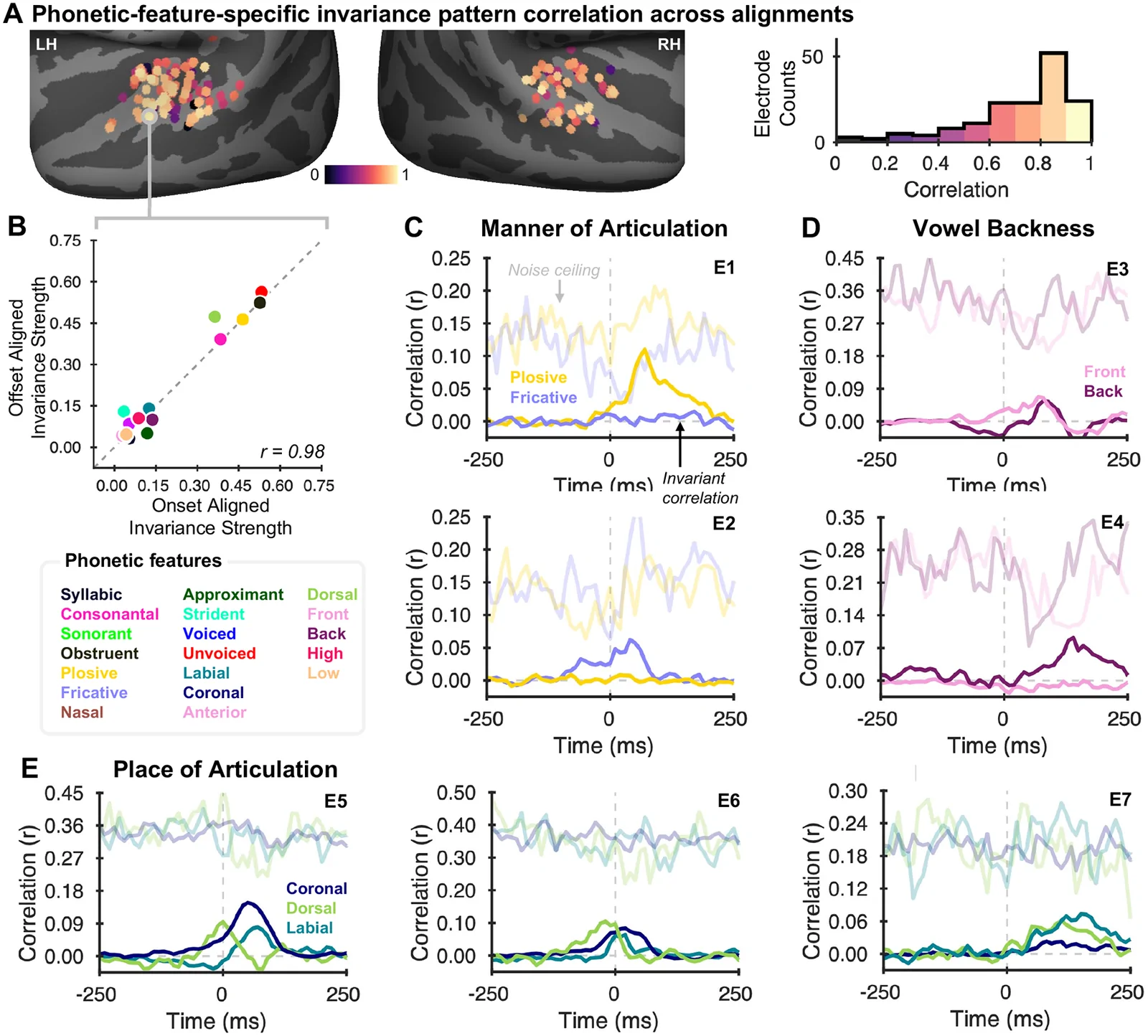
Phonetic-feature-specific invariance using responses aligned to phoneme offset. **(A)** Cortical map of correlation between phonetic-feature-specific invariance patterns across alignment choices; right shows distribution across electrodes. **(B)** Correspondence of phonetic-feature-specific invariance pattern across alignment choices for an example electrode. Each point reflects invariance strength for one phonetic feature estimated from responses aligned to phoneme onset (x-axis) or offset (y-axis). **(C)** Invariant correlation (saturated) and noise ceiling (desaturated) time courses measured from responses aligned to phoneme offset for example electrodes showing differential responses for phonetic features related to manner of articulation. **(D)** Same as (C), for vowel backness. **(E)** Same as (C), for place of articulation.

**Figure A3:**
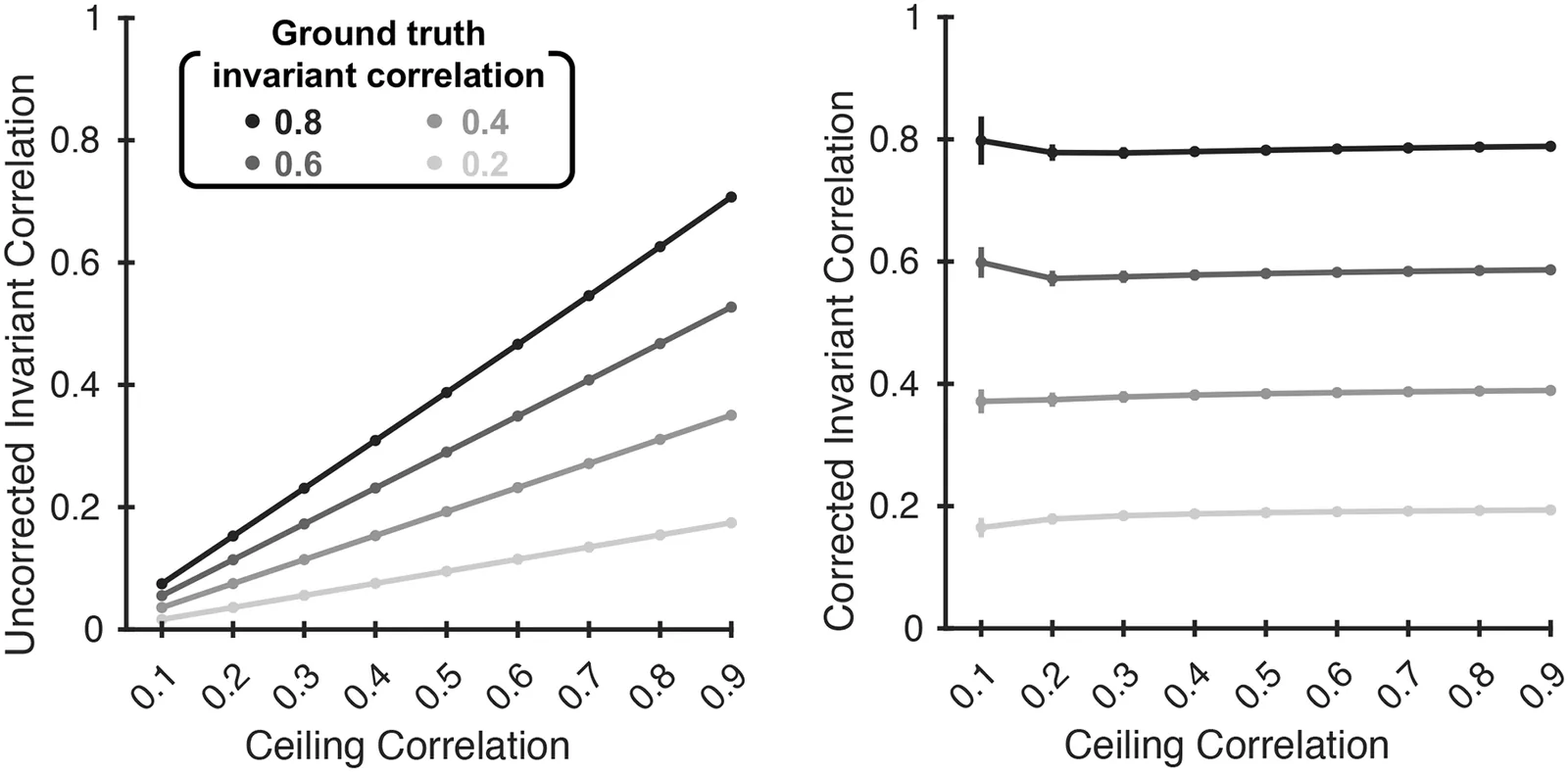
Noise correction simulation results. **(A)** Uncorrected invariant correlation is plotted as a function of the ceiling correlation (noise ceiling) for different ground truth invariant correlation values. Without noise correction, the invariant correlation is attenuated by measurement noise. Note lower ceiling correlation corresponds to greater noise. **(B)** Corrected invariant correlation is plotted as a function of the ceiling correlation (noise ceiling) for different ground truth invariant correlation values. With noise correction, the invariant correlation is robust to measurement noise and accurately recovers ground truth invariant correlation values.

**Figure A4:**
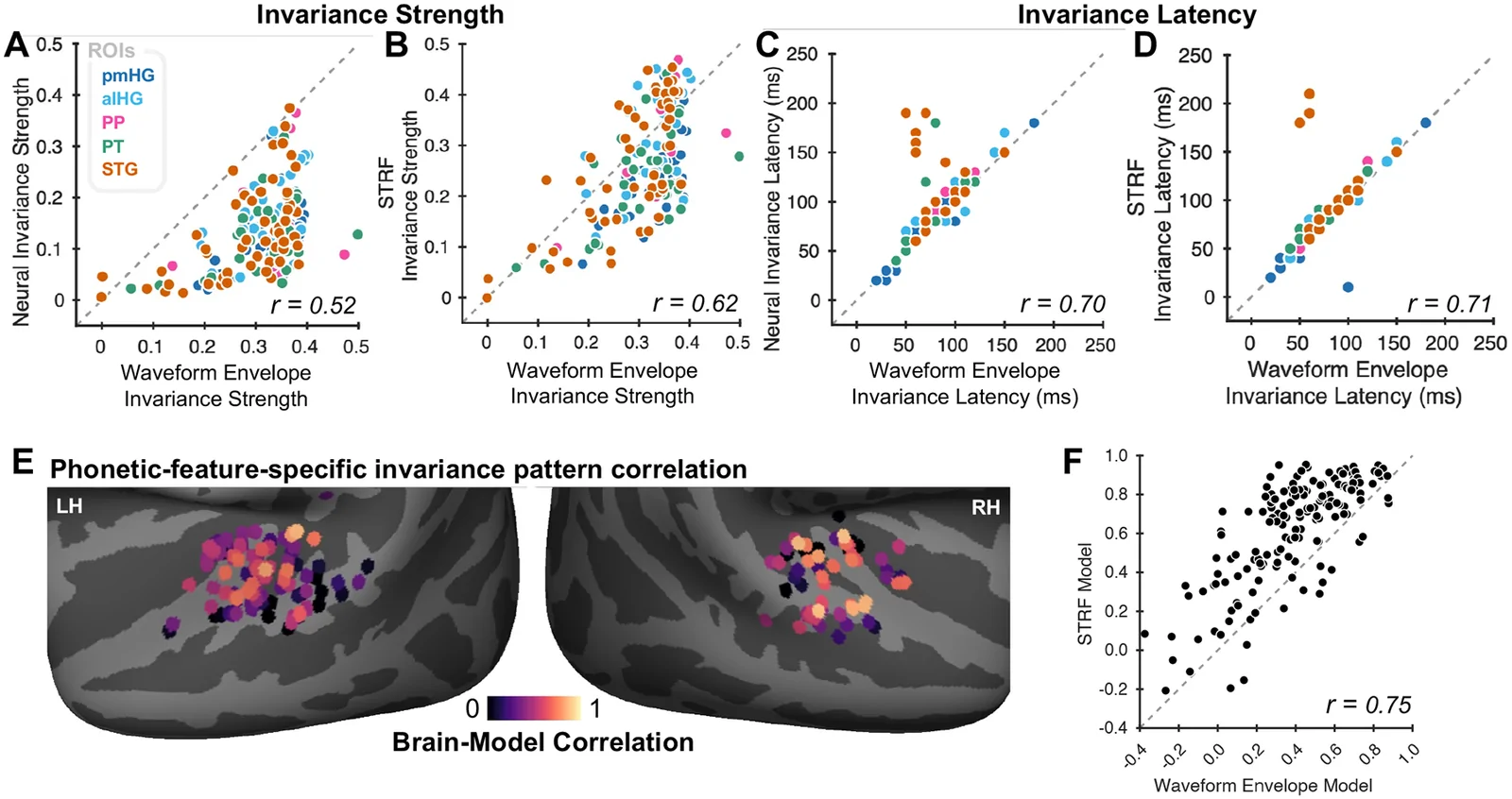
Comparing invariance in computational models and neural data. **(A)** Invariance strength measured from waveform envelope model predictions (x-axis) vs. neural data (y-axis) across electrodes (colored by anatomical ROI). **(B)** Invariance strength measured from waveform envelope model predictions (x-axis) vs. STRF model predictions (y-axis) across electrodes (colored by anatomical ROI). **(C)** Same as (A) for invariance latency. **(D)** Same as (B) for invariance latency. **(E)** Cortical map showing correlation of phonetic-feature-specific invariance patterns between waveform envelope model and neural data for each electrode. **(F)** Comparison of brain-model correlations (quantity plotted in (E)) between encoding models (x-axis, waveform envelope model; y-axis STRF model) for each electrode.

**Figure A5:**
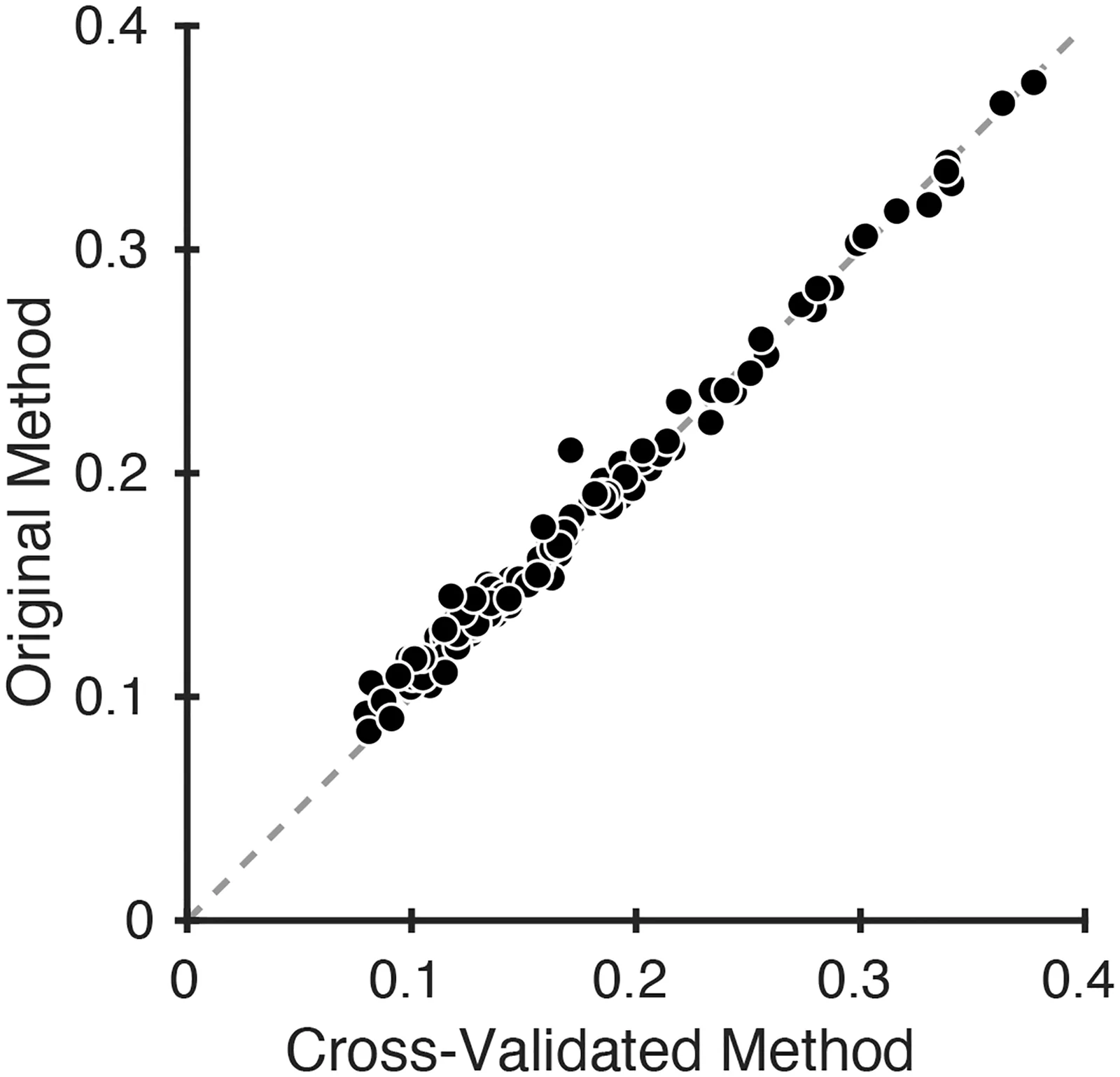
Comparison of methods to robustly estimate invariance strength. Both methods for estimating invariance strength via identifying peaks in the invariant correlation time course produce highly similar results.

## Appendix

### Invariant correlation

This section provides a simple statistical interpretation of the invariant correlation. Under a generative model in which neural responses contain both a class-level signal and instance-specific variability, we show that the invariant correlation measures the proportion of variance attributable to the class-level signal.

Suppose there are a total of K classes (e.g., phoneme categories), and for each class c∈{1,…,K}, there are a total of $n_{c}$ unique instances of that class. There are a total of $N=\sum_{c}n_{c}$ instances/stimuli. Let i index each instance and let c(i) return the class label of stimulus i.

Let $x_{i}=s_{c(i)}+u_{i}$ be the response to stimulus i where $s_{c(i)}$ is a class-level signal shared across all instances of class c and $u_{i}$ is a unique signal for each instance (i.e., is independent across instances). Because the invariant correlation is computed separately for each time point, we focus only on a single time point here for illustrative purposes.

Assume $s_{c(i)}\sim 𝒩\left(0,\sigma_{s}^{2}\right),u_{i}\sim 𝒩\left(0,\sigma_{u}^{2}\right)$, and everything is independent. You can think of $\sigma_{s}^{2}$ as the between-class variance and $\sigma_{u}^{2}$ as the within-class variance.

Now we can collect responses to all stimuli into a stimulus response vector x. Let π be a permutation of {1,…,N} such that c(π(i))=c(i) and π(i)≠i. Define a permuted response vector $x^{\prime }$ such that $x_{i}^{\prime }=x_{\pi (i)}$.

The invariant correlation is given by

$$\rho_{\text{inv}}=\text{Corr}\left(x,x^{\prime }\right)=\frac{\text{Cov}\left(x,x^{\prime }\right)}{\sqrt{\text{Var}(x)\text{Var}\left(x^{\prime }\right)}}$$

Because both vectors have the same distribution, $\text{Var}(x)=\text{Var}\left(x^{\prime }\right)$ giving

$$\rho_{\text{inv}}=\frac{\text{Cov}\left(x,x^{\prime }\right)}{\text{Var}(x)}$$

Now we can expand the numerator. Since all variables are zero-mean, we have

$$\text{Cov}\left(x,x^{\prime }\right)=\frac{1}{N}\sum_{i}E\left[x_{i}x_{i}^{\prime }\right]$$

Now expand the product using

$$x_{i}=s_{c(i)}+u_{i}$$

and

$$x_{i}^{\prime }=x_{\pi (i)}=s_{c(\pi (i))}+u_{\pi (i)}=s_{c(i)}+u_{\pi (i)}$$

to get

$$x_{i}x_{i}^{\prime }=\left(s_{c(i)}+u_{i}\right)\left(s_{c(i)}+u_{\pi (i)}\right)\;=s_{c(i)}^{2}+s_{c(i)}u_{i}+s_{c(i)}u_{\pi (i)}+u_{i}u_{\pi (i)}$$

Since the variables are independent and zero-mean, using the linearity of the expectation, we have

$$E\left[x_{i}x_{i}^{\prime }\right]=E\left[s_{c(i)}^{2}+s_{c(i)}u_{i}+s_{c(i)}u_{\pi (i)}+u_{i}u_{\pi (i)}\right]\;=E\left[s_{c(i)}^{2}\right]+E\left[s_{c(i)}u_{i}\right]+E\left[s_{c(i)}u_{\pi (i)}\right]+E\left[u_{i}u_{\pi (i)}\right]\;=\sigma_{s}^{2}+0+0+0\;=\sigma_{s}^{2}$$

Thus we see $\text{Cov}\left(x,x^{\prime }\right)=\sigma_{s}^{2}$.

Now we turn to the denominator. Similar to the calculation above, we have

$$\text{Var}(x)=\frac{1}{N}\sum_{i}E\left[x_{i}^{2}\right]$$

and

$$E\left[x_{i}^{2}\right]=E\left[s_{c(i)}^{2}+2s_{c(i)}u_{i}+u_{i}^{2}\right]\;=E\left[s_{c(i)}^{2}\right]+E\left[2s_{c(i)}u_{i}\right]+E\left[u_{i}^{2}\right]\;=\sigma_{s}^{2}+0+\sigma_{u}^{2}\;=\sigma_{s}^{2}+\sigma_{u}^{2}$$

Thus we see $\text{Var}(x)=\sigma_{s}^{2}+\sigma_{u}^{2}$.

Substituting back into the expression for the invariant correlation, we get

ρinv=σs2σs2+σu2

Thus, under this generative model, the expected invariant correlation equals the proportion of variance attributable to the class-level signal. For finite datasets, the empirical invariant correlation will fluctuate around this value because the covariance and variance terms are estimated from a limited number of observations.

### Invariant correlation along a multivariate projection

The derivation above considers a single scalar response. Here we extend the result to multivariate neural responses and show how the invariant correlation behaves after projecting population activity onto an arbitrary linear dimension.

Instead of a single scalar $x_{i}$, let the response to stimulus i be a population vector ${\text{x}}_{i}\in R^{D}$ where D is the number of population dimensions (e.g., electrodes) such that we can write

$${\text{x}}_{i}={\text{s}}_{c(i)}+{\text{u}}_{i}$$

We can stack all N stimulus responses to form the matrix $X\in R^{N\times D}$ where each row is the response for a specific stimulus. Consider the projection of this matrix onto the direction $\text{v}\in R^{D}$ to obtain the vector of scalar responses $y\in R^{N}$ for each stimulus

y=Xv

For a single stimulus instance i, the projected scalar response $y_{i}$ is given by

$$y_{i}={\text{x}}_{i}^{⊤}\text{v}\;={\left({\text{s}}_{c(i)}+{\text{u}}_{i}\right)}^{⊤}\text{v}\;={\text{s}}_{c(i)}^{⊤}\text{v}+{\text{u}}_{i}^{⊤}\text{v}\;=s_{c(i)}^{*}+u_{i}^{*}$$

Thus, you can see that y has the same form as x from above. So to find the invariant correlation for y and $y^{\prime }$, we simply need to compute the variance for the projected terms $s_{c(i)}^{*}$ and $u_{i}^{*}$. For $s_{c(i)}^{*}$ we have

$$\text{Var}\left(s_{c(i)}^{*}\right)=\text{Var}\left({\text{s}}_{c(i)}^{⊤}\text{v}\right)\;=E\left[{\left({\text{s}}_{c(i)}^{⊤}\text{v}\right)}^{2}\right]\;=E\left[{\left({\text{s}}_{c(i)}^{⊤}\text{v}\right)}^{⊤}\left({\text{s}}_{c(i)}^{⊤}\text{v}\right)\right]\;=E\left[{\text{v}}^{⊤}{\text{s}}_{c(i)}{\text{s}}_{c(i)}^{⊤}\text{v}\right]\;={\text{v}}^{⊤}E\left[{\text{s}}_{c(i)}{\text{s}}_{c(i)}^{⊤}\right]\text{v}\;={\text{v}}^{⊤}\Sigma_{s}\text{v}$$

where $\Sigma_{s}$ is the D×D between-class covariance matrix. Similarly, for $u_{i}^{*}$, we have $\text{Var}\left(u_{i}^{*}\right)={\text{v}}^{⊤}\Sigma_{u}\text{v}$ where $\Sigma_{u}$ is the D×D within-class covariance matrix. Substituting this into our expression for the invariant correlation, we get

ρinvv=v⊤Σsvv⊤Σsv+v⊤Σuv

Thus, the expected invariant correlation along projection v is determined by the fraction of projected variance attributable to between-class structure.

### Relationship with decoding

The invariant correlation is closely related to classical measures of class discriminability. In this section, we show that the invariant correlation can be written as a simple transformation of the Fisher criterion used in linear discriminant analysis.

A standard way to construct a linear decoder is by using linear discriminant analysis (LDA). In LDA, one seeks to find a projection v to maximize the Fisher criterion J(v) which quantifies the discriminability of classes as the ratio of between-class variance to within-class variance

$$J(\text{v})=\frac{{\text{v}}^{⊤}\Sigma_{s}\text{v}}{{\text{v}}^{⊤}\Sigma_{u}\text{v}}$$

Intuitively, when the numerator is large relative to the denominator, then the between-class variance will dominate, making the classes highly separable. You can think of this ratio as a signal-to-noise ratio where the “signal” comes from the between-class variance and the noise comes from the within-class variance. When the within-class variance is small, then the instances for each class will tightly cluster together, making decoding better (since overlap between classes is reduced) and increasing invariance (since different instances of the same class have very similar responses).

We can write the invariant correlation along the projection v in terms of the Fisher criterion as follows

$$\rho_{\text{inv}}(\text{v})=\frac{{\text{v}}^{⊤}\Sigma_{s}\text{v}}{{\text{v}}^{⊤}\Sigma_{s}\text{v}+{\text{v}}^{⊤}\Sigma_{u}\text{v}}\;=\frac{\frac{{\text{v}}^{⊤}\Sigma_{s}\text{v}}{{\text{v}}^{⊤}\Sigma_{u}\text{v}}}{\frac{{\text{v}}^{⊤}\Sigma_{s}\text{v}}{{\text{v}}^{⊤}\Sigma_{u}\text{v}}+1}$$

Thus, we obtain

ρinvv=JvJv+1

At the population level, the invariant correlation is a bounded, monotonic function of the Fisher criterion. Consequently, the projection that maximizes the Fisher criterion also maximizes the expected invariant correlation under this model. For finite datasets, empirical estimates of discriminability and invariant correlation need not obey this relationship exactly because both quantities are subject to sampling variability.

### Noise correction

In practice, the neural responses we record contain measurement noise that leads to a reduction in both the invariant correlation and discriminability. Thus, we seek to estimate and correct for this noise to obtain unbiased estimates of the true invariant correlation. We do so using the standard correction for attenuation.

As in previous sections, we consider the scalar case but now add noise such that the measured response x to stimulus i is given by

$$x_{i}=s_{c(i)}+u_{i}+\epsilon$$

with $s_{c(i)}\sim 𝒩\left(0,\sigma_{s}^{2}\right),u_{i}\sim 𝒩\left(0,\sigma_{u}^{2}\right)$, and $\epsilon \sim 𝒩\left(0,\sigma_{\epsilon }^{2}\right)$ all independent.

Again, as before, we can concatenate responses to all stimuli into a vector x and create a permuted version $x^{\prime }$ to compute the invariant correlation. In the absence of noise, we would have

ρinv, true=σs2σs2+σu2

However, in the presence of noise, the observed invariant correlation is given by

ρinv, obs=σs2σs2+σu2+σϵ2

Note this is because the denominator is the variance of the response x, but since each component of the response is independent, this is just sum of the variances.

Thus you can see that increasing noise variance $\sigma_{\epsilon }^{2}$ will drive the invariant correlation down. We can correct for this attenuation by estimating then dividing by a ceiling correlation where the ceiling correlation is simply the correlation of the responses across repeated measurements $x^{\left(1\right)}$ and $x^{\left(2\right)}$.

$$\rho_{\text{ceiling}}=\text{Corr}\left(x^{\left(1\right)},x^{\left(2\right)}\right)=\frac{\text{Cov}\left(x^{\left(1\right)},x^{\left(2\right)}\right)}{\sqrt{\text{Var}\left(x^{\left(1\right)}\right)\text{Var}\left(x^{\left(2\right)}\right)}}$$

Like before, since $x^{\left(1\right)}$ and $x^{\left(2\right)}$ have the same distribution, we get

$$\rho_{\text{ceiling}}=\frac{\text{Cov}\left(x^{\left(1\right)},x^{\left(2\right)}\right)}{\text{Var}(x)}$$

The denominator is the same as what we saw previously for the observed invariant correlation. However, the numerator is slightly different. For the ith stimulus, the responses across different repetitions are given by $x_{i}^{\left(1\right)}=s_{c(i)}+u_{i}+\epsilon^{\left(1\right)}$ and $x_{i}^{\left(2\right)}=s_{c(i)}+u_{i}+\epsilon^{\left(2\right)}$. Because the class-level signal $\left(s_{c(i)}\right)$ and the instance-specific contribution $\left(u_{i}\right)$ are fixed properties of the stimulus, they are identical across repeats. Only the measurement noise ϵ varies across repetitions.

Taking the expectation of their product we get

$$E\left[x_{i}^{\left(1\right)}x_{i}^{\left(2\right)}\right]=E\left[\left(s_{c(i)}+u_{i}+\epsilon^{\left(1\right)}\right)\left(s_{c(i)}+u_{i}+\epsilon^{\left(2\right)}\right)\right]\;=E\left[{\left(s_{c(i)}+u_{i}\right)}^{2}+\left(s_{c(i)}+u_{i}\right)\epsilon^{\left(2\right)}+\right.\left.\left(s_{c(i)}+u_{i}\right)\epsilon^{\left(1\right)}+\epsilon^{\left(1\right)}\epsilon^{\left(2\right)}\right]\;=E\left[{\left(s_{c(i)}+u_{i}\right)}^{2}\right]+E\left[\left(s_{c(i)}+u_{i}\right)\epsilon^{\left(2\right)}\right]+E\left[\left(s_{c(i)}+u_{i}\right)\epsilon^{\left(1\right)}\right]+E\left[\epsilon^{\left(1\right)}\epsilon^{\left(2\right)}\right]\;=\left(\sigma_{s}^{2}+\sigma_{u}^{2}\right)+0+0+0\;=\sigma_{s}^{2}+\sigma_{u}^{2}$$

Similar to previous calculations, we thus have $\text{Cov}\left(x^{\left(1\right)},x^{\left(2\right)}\right)=\sigma_{s}^{2}+\sigma_{u}^{2}$

Thus the ceiling correlation can be written as

ρceiling=σs2+σu2σs2+σu2+σϵ2

Finally, taking the ratio of the observed invariant correlation to the ceiling correlation yields the true invariant correlation

$$\frac{\rho_{\text{inv, obs}}}{\rho_{\text{ceiling}}}=\frac{\frac{\sigma_{s}^{2}}{\sigma_{s}^{2}+\sigma_{u}^{2}+\sigma_{\epsilon }^{2}}}{\frac{\sigma_{s}^{2}+\sigma_{u}^{2}}{\sigma_{s}^{2}+\sigma_{u}^{2}+\sigma_{\epsilon }^{2}}}=\frac{\sigma_{s}^{2}}{\sigma_{s}^{2}+\sigma_{u}^{2}}=\rho_{\text{inv, true}}$$

### Methods

#### Participants and data collection

Data presented in this paper were collected from 31 patients undergoing treatment for intractable epilepsy at the University of Rochester Medical Center (URMC; N=18) and University of Iowa Hospitals and Clinics (UIHC; N=13) (18 male, 13 female; mean age 39.1 years, sd = 10.3 years). Stereotactic depth electrodes were implanted to localize epileptogenic zones and delineate these zones from eloquent cortical areas before brain resection. All participants gave informed written consent to participate in the study, which was approved by the Institutional Review Boards of URMC and UIHC. URMC patients were compensated $35 per hour. UIHC patients were not compensated because of Institutional Review Board prohibition.

#### Stimuli and experimental design

Speech stimuli were comprised of sentences from the Harvard IEEE corpus (“IEEE Recommended Practice for Speech Quality Measurements”, 1969). There were 15 sentences, each with 5–7 words (all but one word was monosyllabic). A greedy algorithm was used to select sentences that were maximally phonemically diverse, as measured by the entropy of the phoneme histogram. Recordings of the chosen Harvard IEEE sentences were obtained from the UW/NU v2 corpus (Panfili et al., 2017). Sentences were spoken by 32 unique talkers (half female), each of whom spoke 1–3 sentences. Individual sentence recordings were resampled to 20 kHz, RMS normalized, and ramped (25 ms, symmetric Hanning window). Phoneme onsets and offsets were obtained from the Praat TextGrids included in the UW/NU v2 corpus (Panfili et al., 2017).

The individual sentence recordings were presented in a randomized order (concatenated together using cross-fading, 25 ms Hanning window) with the constraint that no sentence could repeat back-to-back. We also created random word sequences, in which the constituent words were excerpted and re-ordered again with the constraint that no word could repeat back-to-back (concatenated using cross-fading). The results were similar for both natural sentence and random word sequences, and thus, we computed our measures across both.

For other ongoing experiments, the speech stimuli for all conditions were presented using three different speech rates (unchanged, $\sqrt{2}$ faster, and $\sqrt{2}$ slower), all of which were highly intelligible. The speech rate was manipulated using time compression/stretching with the vocoder STRAIGHT (Kawahara, 2006). The results were similar for all speech rates tested, and thus, we computed our measures across all speech rates.

In total, there were 6 stimulus sequences (corresponding to sentences and random word sequences, each presented at three rates; the sequences varied in duration between 62 to 125 seconds). Each sequence was presented twice per experimental session, and some participants completed multiple sessions. To encourage sustained attention to the speech stimuli, participants were asked to press a button when they heard a target word (“green”).

#### Intracranial data preprocessing

Our preprocessing pipeline was similar to other intracranial EEG studies (Norman-Haignere et al., 2022). Electrode responses were common-average referenced to the grand mean across electrodes from each participant. We excluded noisy electrodes from the common-average reference by detecting anomalies in the 60 Hz power band (measured using an infinite impulse response filter with a 3 dB down bandwidth of 0.6 Hz, implemented using MATLAB’s iirpeak.m function). Specifically, we excluded electrodes whose 60 Hz power exceeded five standard deviations of the median across electrodes. Because the standard deviation is itself sensitive to outliers, we estimated the standard deviation using the central 20% of samples (dividing the span of the central 20% of samples by that which would be expected from a unit-variance Gaussian). Next, we applied a high-pass filter with a very low cutoff (using a fourth-order Butterworth filter with 3 dB down cutoff of 0.5 Hz, implemented using fdesign.highpass in MATLAB). After common-average referencing, we used a notch filter to remove harmonics and fractional multiples of the 60 Hz noise (60, 120, 180, and 240 Hz, using an infinite impulse response notch filter with a 3 dB down bandwidth of 1 Hz; the filter was applied forward and backward; implemented using MATLAB’s iirnotch.m function).

Analyses were performed on the broadband gamma power response time course of each electrode. We computed broadband gamma power by measuring the envelope of the pre-processed signal filtered between 70 and 140 Hz (implemented using a sixth-order Butterworth filter with 3 dB down cutoffs of 70 and 140 Hz; the filter was applied forward and backward; envelopes were measured using the absolute value of the analytic signal, computed using the Hilbert transform; implemented using fdesign.bandpass in MATLAB).

As is standard, we time-locked the intracranial recordings to the stimuli by detecting a series of pulses at the start of each stimulus that were recorded synchronously with the intracranial data. We used the stereo jack on the experimental laptop to send audio and pulses on separate channels. The audio on one channel was used to play sounds to participants, and the pulses on the other were sent to the recording rig. Sounds were played through a Genelec 80101A speaker. Responses were converted to units of percent signal change relative to silence by subtracting and then dividing the response of each electrode by the average response during the 500 ms before each stimulus.

#### Electrode localization

We used the FieldTrip toolbox (Oostenveld et al., 2011) to localize electrodes as bright spots on a post-operative computer tomography (CT) image. The post-operative CT was aligned to a high-resolution, pre-operative MRI that was undistorted by electrodes. Each electrode was projected onto the cortical surface computed by Freesurfer (Dale et al., 1999) from the pre-operative MRI, excluding electrodes greater than 10 mm from the surface. We used a correction procedure from another study (Norman-Haignere et al., 2022) to correct gross-scale errors by encouraging points that are nearby in 3D space but far apart on the 2D cortical surface (e.g., two abutting gyri) to preferentially be localized to regions where sound responses are common.

#### Electrode selection

We selected electrodes with a reliable broadband gamma response to the speech stimuli. Specifically, we measured the test-retest correlation of each electrode’s response to each stimulus (using odd versus even repetitions for participants who heard more than two repetitions), and then averaged the correlation values across stimuli to obtain a single reliability measure for each electrode. The significance of this measure was determined using a nonparametric permutation test (permuting 500 ms segments of neural recording 1000 times). We used a Gaussian fit to the distribution of permuted correlation coefficients to compute small p-values and selected only highly significant electrodes $\left(p<10^{-5}\right)$ above a minimum test-retest correlation baseline (r=0.05) for further analysis. Based on these criteria, we identified 250 electrodes that showed a reliable response. For these electrodes, we computed the noise ceiling and applied an additional criterion, retaining only electrodes with an average noise ceiling greater than 0.05 between 0 and 500 ms post phoneme onset, yielding a total of 183 electrodes (from 25 unique patients) for our invariance analyses.

#### Quantifying invariance to acoustic variation

We quantified invariance using the invariant correlation as described in the main text. However, we note that there are many possible permutations of stimuli that satisfy the pairing criteria (same phoneme identity from a different word spoken by a different talker) used to compute the invariant correlation. To ensure robustness of our results, we computed the invariant correlation for 100 random permutations and averaged the resulting time courses.

#### Invariance strength and latency

To extract summary metrics from the noisy invariant correlation time course, we first smoothed it using a Gaussian kernel (50 ms full-width-half-max) and identified the time and value of the maximum. We normalized this peak value by the noise ceiling at the corresponding time point to obtain the invariance strength. Specifically, we smoothed the noise ceiling using the same kernel and evaluated it at the time of the peak. We note this peak-based measure can be subject to upward bias, but we confirmed that such bias is minimal in our data as our results are unchanged when peaks are selected using cross-validation (Fig. A5; correlation between magnitudes estimated with each approach r=0.99, p<0.001). Briefly, our cross-validated procedure involved randomly splitting data in half, computing invariance time courses for each half, identifying the peak time (as above) in one half and extracting the magnitude at the corresponding time in the other half, then averaging the resulting magnitudes across halves and 100 random splits. Since our approach showed no evidence of an upward bias, we chose to report results using peaks identified without cross-validation to avoid additional computational complexity. We also note that our goal is to illustrate the invariant correlation method rather than make definitive claims about absolute magnitudes. To test whether the peak invariant correlation was significantly greater than chance, we generated a null distribution by computing the invariant correlation 1,000 times with randomly permuted rows of the response matrices. We applied the same smoothing and peak identification procedure to each permutation, yielding a null distribution of peak values. For electrodes whose observed peak exceeded chance (p<0.05), we defined the invariance latency as the timing of the peak.

#### Noise correction simulation

To validate our noise correction procedure, we ran simulations using synthetic data with known levels of invariance and confirmed that our procedure accurately recovered the ground-truth invariance across noise levels. Because the invariant correlation is computed independently at each time point, we conducted our simulations corresponding to responses at a single time point. We generated responses for multiple stimulus classes (e.g., phoneme classes), each with multiple instances, where each response was composed of a class-level signal shared across all instances of that class and an instance-specific component unique to each instance. We then added measurement noise to these responses. The relative variances of these components were set to produce a range of ground-truth invariant correlations, while the noise variance was adjusted to yield a range of noise ceiling values.

#### Phonetic features

We analyzed invariance with respect to 19 phonetic features based on standard phonological classifications: syllabic, consonantal, sonorant, obstruent, plosive, fricative, nasal, approximant, strident, voiced, unvoiced, labial, coronal, anterior, dorsal, front, back, high, low. These features have been widely used to characterize structure in auditory cortical representations of speech (Hamilton et al., 2021; Keshishian et al., 2023; Mesgarani et al., 2014; Yi et al., 2019).

#### Spectrotemporal receptive field encoding model

We tested whether our findings could be explained by standard spectrotemporal receptive field (STRF) models. STRFs were fit by mapping a cochleagram representation of sound onto the response of each electrode to the speech stimuli used in this experiment. Cochleagrams are similar to spectrograms but are computed using filters whose frequency characteristics are intended to coarsely match those in the cochlea (amplification is captured using a compressive power law applied to subband envelopes) (see Norman-Haignere and McDermott, 2018 for details of cochleagram computation). Following common practice, STRFs were estimated using regularized regression against lagged cochleagram features, producing a time lag × frequency weight matrix for each electrode (51 lags, spanning 0–500 ms). Cross-validation was used to prevent overfitting with the first and second half of each sequence used for training and testing. The ridge penalty was selected using an inner cross-validation loop within the train set. We then applied our invariance analyses to the predictions in test data from the fit STRF model.

#### Waveform envelope encoding model

Waveform envelopes were computed by squaring the waveform, convolving with a Gaussian kernel (25 ms FWHM), taking the square root, downsampling to 100 Hz, and then applying power compression (exponent of 0.3). The smoothing kernel and compression exponent were selected empirically as those that maximized the prediction accuracy of the data. We used ridge regression to fit an encoding model using the waveform envelope as features then applied our invariance analyses to the predictions in test data from the fit waveform envelope model. The procedure was identical to that described above for the STRF encoding model.
